# The In Vitro Antibacterial Activity of Phytogenic and Acid-Based Eubiotics against Major Foodborne Zoonotic Poultry Pathogens

**DOI:** 10.3390/ani14111611

**Published:** 2024-05-29

**Authors:** Konstantinos Kiskinis, Tilemachos Mantzios, Vangelis Economou, Evanthia Petridou, Anestis Tsitsos, Apostolos Patsias, Ioanna Apostolou, Georgios A. Papadopoulos, Ilias Giannenas, Paschalis Fortomaris, Vasilios Tsiouris

**Affiliations:** 1 Unit of Avian Medicine, Clinic of Farm Animals, School of Veterinary Medicine, Aristotle University of Thessaloniki, 54627 Thessaloniki, Greece; mantzios@vet.auth.gr (T.M.); biltsiou@vet.auth.gr (V.T.); 2 Laboratory of Food Animal Hygiene and Veterinary Public Health, School of Veterinary Medicine, Aristotle University of Thessaloniki, 54124 Thessaloniki, Greece; boikonom@vet.auth.gr (V.E.); tsitanes@vet.auth.gr (A.T.); 3 Laboratory of Microbiology and Infectious Diseases, School of Veterinary Medicine, Aristotle University of Thessaloniki, 54124 Thessaloniki, Greece; epetri@vet.auth.gr; 4 Agricultural Poultry Cooperation of Ioannina “PINDOS”, Rodotopi, 45500 Ioannina, Greece; apatsias@pindos-apsi.gr; 5 National Reference Laboratory (NRL) for Campylobacter, Veterinary Laboratory of Ioannina, 45221 Ioannina, Greece; apostolouioanna1@gmail.com; 6 Laboratory of Animal Science, School of Veterinary Medicine, Aristotle University of Thessaloniki, 54124 Thessaloniki, Greece; geopaps@vet.auth.gr (G.A.P.); fortomap@vet.auth.gr (P.F.); 7 Laboratory of Nutrition, School of Veterinary Medicine, Aristotle University of Thessaloniki, 54124 Thessaloniki, Greece; igiannenas@vet.auth.gr

**Keywords:** poultry, zoonotic bacteria, phytogenic additives, acid-based eubiotics, antimicrobial activity, minimum inhibitory concentration (MIC)

## Abstract

**Simple Summary:**

Antibiotic-resistant pathogens from overuse or misuse of antibiotics in livestock and poultry to treat human and animal diseases have become a global threat. Therefore, alternatives to the use of antibiotics have become imperative. The aim of the study was the in vitro antibacterial investigation of eight drinking water additives based on essential oils phytogenics (Phyto CSC Liquide B, AEN 350 B Liquid), acid-based eubiotics (Salgard liquid, Intesti-Flora) and blends of essential oils and organic acids (ProPhorce^TM^ SA Exclusive, Herbal acid, Rigosol-N and Eubisan 3000) against Gram-negative bacteria such as *Campylobacter* spp., *Escherichia coli*, *Salmonella* Typhimurium and Gram-positive bacteria such as *Staphylococcus aureus* and *Listeria* spp. The results showed that most of the tested products had promising antibacterial activity. Specifically, the products categorized as “Blends of essential oils and organic acids” performed the highest antibacterial capacity, followed by the “acid-based eubiotics”, while products within the “essential oil-based phytogenics” category performed the lowest antibacterial capacity. Concluding, phytogenic and acid-based eubiotics, as well as their combination, could be good candidates for pathogen control on poultry farms and for the reduction of antimicrobial resistance.

**Abstract:**

The aim of the study was to investigate in vitro the antibacterial activity of 8 commercial drinking water additives against major zoonotic poultry pathogens (*Campylobacter* spp., *Escherichia coli*, *Salmonella* Typhimurium, *Staphylococcus aureus* and *Listeria* spp.). We tested two essential oil-based phytogenics (Phyto CSC Liquide B, AEN 350 B Liquid), two acid-based eubiotics (Salgard^®^ liquid, Intesti-Flora), and four blends of essential oils and organic acids (ProPhorce^TM^ SA Exclusive, Herbal acid, Rigosol-N and Eubisan 3000). The antibacterial activity was determined by estimating the minimum inhibitory concentration (MIC) using a microdilution method. The MICs of the products against *Campylobacter* spp. ranged from 0.071% to 0.568% *v*/*v*, in which Herbal acid, a blend rich in lactic and phosphoric acids, also containing thyme and oregano oils, exhibited the highest efficacy (MIC: 0.071% *v*/*v*) against all the tested strains. The MICs of the tested products against *Escherichia coli* ranged between 0.071% and 1.894% *v*/*v*. Specifically, the MIC of Rigosol-N, a blend of high concentrations of lactic and acetic acid, was 0.142% *v*/*v* for both tested strains, whereas the MICs of Intesti-Flora, a mixture rich in lactic and propionic acid, ranged from 0.284% to 0.568% *v*/*v*. The MICs of the products against *Salmonella* Typhimurium were between 0.095% and 1.894% *v*/*v*. Specifically, the MIC of Eubisan 3000, a blend rich in oregano oil, was 0.284% *v*/*v*. The MICs against *Staphylococcus aureus* were between 0.142% and 9.090% *v*/*v*. The MICs of Phyto CSC Liquide B, which is rich in *trans*-cinnamaldehyde, were between 3.030% and 9.090% *v*/*v*, showing the highest MIC values of all tested products. Finally, the MIC values of the tested commercial products against *Listeria* spp. were 0.095% to 3.030% *v*/*v*. The MICs of ProPhorce^TM^ SA Exclusive, a highly concentrated blend of formic acid and its salts, were 0.095–0.142% *v*/*v* against *Listeria* spp., while the MICs of AEN 350 B Liquid were between 0.284% and 1.894% exhibiting high *Listeria* spp. strain variability. In conclusion, all the selected commercial products exhibited more or less antibacterial activity against pathogenic bacteria and, thus, can be promising alternatives to antibiotics for the control of zoonotic poultry pathogens and the restriction of antimicrobial-resistant bacteria.

## 1. Introduction

Poultry meat is a significant source of high-quality animal protein without any societal, cultural, or religious constraints. According to OECD-FAO 2023, it is estimated that 41% of global human protein consumption is predicted to originate from poultry meat by 2032 [1]. However, higher production requires a greater concentration of poultry in crowded conditions on large intensive livestock farms. This may lead to a rise in the incidence and spread of viral and bacterial diseases, including zoonoses, according to EFSA-EMA 2017 [2].

Currently, the most prevalent and noteworthy foodborne zoonotic poultry pathogens are *Campylobacter* spp., *Salmonella* spp., *E. coli*, *Staphylococcus* spp., and *Listeria* spp. [3,4]. In terms of public health, these pathogenic poultry bacteria may induce fever, stomach cramps, diarrhea, and nausea, whereas infections by *Listeria* spp. can also induce stiff neck, confusion, loss of balance, convulsions, hospitalization, and fatality [5,6,7]. Poultry products are among the most common vehicles for human transmission of *Salmonella* spp. and *Campylobacter* spp., with the latter accounting for the majority of bacterial gastroenteritis cases in Europe [8]. Additionally, human intake of undercooked, contaminated meat, as well as inadequate storage and sanitary practices across the food chain, have been associated with *E. coli* and *S. aureus* infections [4,9,10]. Moreover, ready-to-eat poultry meat products can easily get contaminated with *Listeria* spp. during processing or prolonged refrigerated storage [11].

In poultry, *Campylobacter* spp., *Salmonella* spp., *E. coli*, *Staphylococcus* spp., and *Listeria* spp. have been associated with little-to-severe clinical signs, gross lesions, productivity loss, and welfare issues, all depending on the infected strain, the type of bird hybrid, immunization level, feed composition, and management practices. For *Campylobacter* spp., infections may lead to diarrhea and mucous-tinted dropping, as well as a decrease in body weight and production, while the prevalence rate in poultry flocks is higher than 50% [4]. On the other hand, *Salmonella* spp. infections are usually more severe, leading to anorexia, watery diarrhea, decreased egg production, and a high mortality rate of 80% or even higher, while the financial losses due to *Salmonella* contamination in poultry products is accounting for USD 2.8 billion annually [12,13]. In addition, avian pathogenic *E. coli* (APEC) infections are frequently correlated with perihepatitis, pericarditis, and airsacculitis, accompanied by septicemia. Furthermore, in the U.S., losses in the broiler sector due to *E. coli* can reach up to USD 40 million per year just from carcass condemnation [14]. Finally, *S. aureus* is usually involved in arthritis, synovitis, chondronecrosis, osteomyelitis, gangrenous dermatitis, subdermal abscesses (bumblefoot), and septicemia, leading to economic losses due to lower productivity [15,16].

In previous decades, the abuse or misuse of antibiotics in poultry has hastened the development of antimicrobial resistance (AMR) in foodborne zoonotic poultry pathogens. Particularly, *Campylobacter* spp. may be resistant to macrolides (erythromycin), penicillins (ampicillin), tetracycline, fluoroquinolones (nalidixic acid), ciprofloxacin, trimethoprim/sulfonamides (trimethoprim/sulfamethoxazole), aminoglycosides (streptomycin and gentamicin) and cephalosporins (cephalothin) [17]. The most common resistance observed in *Salmonella* is against fluoroquinolones (nalidixic acid), β-lactams (ampicillin, amoxicillin/clavulanic acid), aminoglycosides (streptomycin) and trimethoprim/sulfonamides (trimethoprim/sulfamethoxazole) [18]. Moreover, *E. coli* has high resistance to fluoroquinolones (nalidixic acid), β-lactams (penicillins) and tetracycline has been recorded [19]. Resistance to antibiotics, including amoxicillin, amoxicillin-clavulanic acid, ampicillin, cefoxitin, kanamycin, penicillin and tetracycline has frequently been reported for *S. aureus* isolated from poultry [16], whereas *Listeria* spp. shows resistant or reduced susceptibility to oxacillin, cefoxitin, cefotaxime, cefepime, rifampicin, ciprofloxacin, enrofloxacin, and nitrofurantoin [20]. Thus, AMR in foodborne zoonotic poultry pathogens is regarded as a serious global health threat.

It was recently suggested that reducing pathogens colonization in poultry at the primary production level may be an important tool to control the incidence of human infections [21]. Among others, water can be an excellent vector for the dissemination of poultry bacterial pathogens [22]. As a result, a variety of natural additives, including phytogenics and acid-based eubiotics, are employed in the drinking water of poultry farms, aiming to reduce or even prevent the spread of infectious diseases [23,24]. Particularly, phytogenics are plant-based additives derived from herbs, spices, and extracts such as essential oils (EOs) [25] that are frequently utilized in poultry nutrition to improve performance and health through their potent antimicrobial, antioxidant, anti-inflammatory, immunomodulatory, and digestion stimulating properties [24]. On the other hand, acid-based eubiotics, such as organic acids and their salts, are naturally occurring substances that possess acidic characteristics [26]. They enhance birds’ health and performance by improving nutrient digestibility, stimulating gastric secretions and the intestinal immune system, reducing pathogen load, and promoting beneficial bacteria [27,28]. Recently, blends of EOs and organic acids have been reported as an efficient formula due to their synergic or additive effect on broilers’ performance and intestinal health [29,30].

Several studies have reported the antimicrobial activity of individual/or blends of essential oils or organic acids [31,32,33,34]. However, the antimicrobial activity of commercial products containing “blends” of the above-mentioned categories is rarely reported. Therefore, the aim of this study was the in vitro investigation of the minimum inhibitory concentration (MIC) of eight commercial water “essential oil-based phytogenics”, “acid-based eubiotics” and “blends of EOs and organic acids” (Phyto CSC Liquide B, AEN 350 B Liquid, Salgard^®^ liquid, Intesti-Flora, ProPhorce^TM^ SA Exclusive, Herbal acid, Rigosol-N and Eubisan 3000) against major foodborne zoonotic poultry pathogens, including *Campylobacter* spp., *E. coli*, *Salmonella* spp., *Staphylococcus* spp., and *Listeria* spp. The commercial products were selected due to their wide application in primary poultry production.

## 2. Materials and Methods

### 2.1. Products under Examination

Eight different commercial products used in drinking water on poultry farms and authorized by the EU were tested for their antimicrobial activity against major foodborne zoonotic poultry pathogens. The products were divided into three categories based on their synthesis:

- Essential oil-based phytogenics: Phyto CSC Liquide B (Phytosynthese, Mozac, France), AEN 350 B Liquid (Phytosynthese, Mozac, France)
- Acid-based eubiotics: Salgard^®^ liquid (Anpario plc, Manton Wood Enterprise Park, Worksop, Nottinghamshire, UK), Intesti-Flora (Kanters, Lieshout, The Netherlands)
- Blends of essential oils and organic acids: ProPhorce^TM^ SA Exclusive (^©^Perstorp, Malmö, Sweden), Herbal acid (Pancosma, Rolle, Switzerland), Rigosol-N (Panaroma EPE, Kilkis, Greece), Eubisan 3000 (MIRAVIT^®^, Münster, Germany)

All the commercial products were in liquid form at ambient temperature. The active compounds of each product and the recommended dosage by the manufacturers are listed in Table 1.

The pH of each concentration of the product in the tested medium was determined to evaluate the effect of it on different concentrations of the commercial products under examination, with the use of a digital pH meter (pH 315i, WTW Wissenschaftlich-Technische Werkstatten, Weilheim, Germany).

### 2.2. Tested Bacterial Strains

Thirteen strains of poultry-associated pathogenic bacteria, 7 gram-negative (G−) and 6 gram-positive (G+), were tested in this study.

Gram-negative strains: *Campylobacter jejuni* S1 (S1: Strain 1) and *Campylobacter coli* S1 were collected between 2018 and 2019 as part of epidemiological surveillance. Both were field isolates of avian and porcine origin, respectively, obtained from the *Campylobacter* strain collection of the Greek National Reference Laboratory for *Campylobacter* in Ioannina, Greece. *Campylobacter jejuni* S2 (S2: Strain 2) was isolated from a commercial poultry slaughterhouse during a routine surveillance program and detected by PCR in 2019. *Escherichia coli* ATCC 25922 [35], a reference strain of clinical provenance, is recommended for antimicrobial susceptibility testing by the Clinical and Laboratory Standards Institute (CLSI) and the European Committee on Antimicrobial Susceptibility Testing (EUCAST), while *Escherichia coli* ATCC 11303 [36], strain obtained from a culture collection, is used as a bacteriophage host and as a model organism for *Escherichia coli* experimental protocols. *Salmonella* Typhimurium DT 120, a pathogenic strain isolated during a salmonellosis outbreak in Denmark after consumption of turkey meat. *Salmonella* Typhimurium U292 is a pathogenic strain isolated during an outbreak in Denmark from a patient with gastrointestinal symptoms [33].

Gram-positive strains: *Staphylococcus aureus* DSM 102262 was isolated from a patient in North Korea, while *Staphylococcus aureus* DSM 25629’s origin is unknown. Both *staphylococci* strains are methicillin and multidrug-resistant pathogens. *Staphylococcus aureus* S1 was a wild strain isolated from a commercial poultry slaughterhouse. *Listeria monocytogenes* Scott A, utilized as a reference pathogenic strain, is a serovar 4b clinical strain that was initially isolated in 1983 during an outbreak in Massachusetts, USA. *Listeria innocua* ATCC 33090 [37] is another reference strain that is used as a quality control strain in microbiological testing. It was originally isolated from a cow brain. Finally, *Listeria monocytogenes* S1, a serotype 1/2a wild strain, was isolated from raw chicken meat [33].

### 2.3. Preparation of the Tested Inoculum

The tested strains were reconstituted from 13% glycerol broth maintained at −80 °C. Fresh cultures were prepared in Brain Heart Infusion broth (BHI, CM1135, Oxoid Ltd., Basingstoke, UK) and incubated according to the conditions specified for each bacteria strain. Particularly, *E*. *coli*, *S.* Typhimurium, *S. aureus*, and *Listeria* spp. were incubated under aerobic conditions at 37 °C for 24 h, whereas *C. jejuni* and *C. coli* were incubated at 42 °C under microaerophilic conditions for 48 h. Each strain was plated on BHI or cation-adjusted Mueller Hinton agar (MHA, B11438, Becton Dickinson, Franklin Lakes, NJ, USA). Finally, *Campylobacter* strains were cultured on MHA + 5% horse blood (Oxoid, CM 129), in microaerophilic conditions, approximately 5% O_2_, 10% CO_2_, and 85% N_2_ (Thermo Scientific™ Oxoid™ CampyGen™ 2.5 L Sachet, Oxoid, Basingstoke, UK). Prior to the preparation of the second culture (working culture), all strain cultures were verified according to their morphological and biochemical characteristics. After inoculation, colonies from each working culture were utilized to generate a 0.5 McFarland suspension in Mueller-Hinton broth (MHB, CM0405, Oxoid Ltd., Basingstoke, UK), using a densitometer (Densimat, Biomerieux, Knutsford, UK) which was then diluted to yield a working solution of 10^6^ CFU of bacteria/mL.

### 2.4. MIC Assay

**Figure 1:**
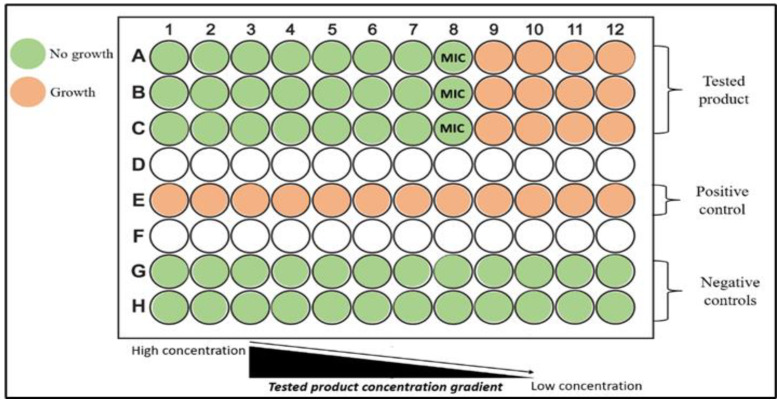
Schematic representation of the microdilution MIC method used to evaluate the antibacterial activity of the commercial drinking water additives against major zoonotic poultry bacteria. The numbers 1–12 referred to the gradient concentrations of the tested products while letters A–C referred to the treatment groups, letter E to positive control, letters G and H to the negative controls, and letters D and F were blank.

The determination of the MIC of the tested products was performed according to CLSI standards M07-A10 [38] and M100-S28 [39], with modifications. Each product was tested against all the selected strains in concentrations ranging from 0.0044 to 9.090% *v*/*v*, using a microdilution MIC method (micro-MIC). Particularly, for each bacterial strain, individual wells in a flat-bottomed 96-well polystyrene microtiter plate (83.3924, TC-Platte 96 Well, Standard, F) were used and filled with cation-adjusted MH broth. Each product was added to the microtiter plates using the two-fold serial dilution method, followed by inoculation of the bacterial strain into each well. This process was applied in triplicate for all the products and all the tested bacterial strains. One strip of wells in each microplate served as the negative control (addition of the sterile culture medium only) and another as the positive control (addition of the bacterial inoculum only), as presented in Figure 1. Each microplate was sealed and incubated for 24 h at 37 °C, under aerobic conditions, apart from the plates containing the *Campylobacter* strains, which were incubated for 48 h at 42 °C under microaerophilic conditions (Thermo Scientific™ Oxoid™ CampyGen™ 2.5 L Sachet, Oxoid, Basingstoke, UK). After incubation, the plates were examined for bacterial growth indicated by turbidity in the broth of the wells. The last dilution that inhibited visual growth was expressed as the MIC of each product against a specific bacterial strain.

### 2.5. Statistical Analysis

The raw data from the MIC measurements were analyzed with SPSS 28.0 (BM SPSS Statistics for Windows^®^, Version 28.0. Armonk, NY, USA: IBM Corp.). The MIC value of each product against each tested pathogen was interpreted as the mean MIC value (% *v*/*v* ± standard deviation) of the individual value of each triplicate.

## 3. Results

**Figure 2:**
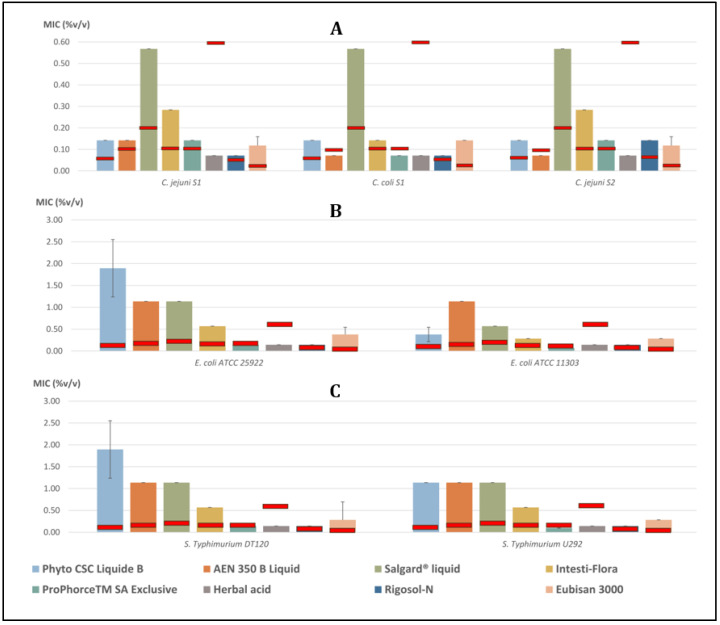
Graphs of recorded MIC values (columns; mean %*v*/*v* ± SD) of the tested commercial products on the tested (**A**) *Campylobacter*, (**B**) *E.coli* and (**C**) *S.* Typhimunium strains.

**Figure 3:**
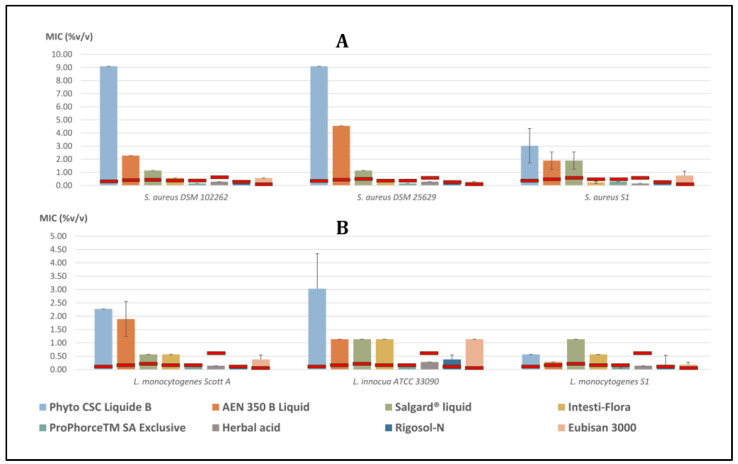
Graphs of recorded MIC values (columns; mean %*v*/*v* ± SD) of the tested commercial products on the tested (**A**) *S. aureus* and (**B**) *Listeria* spp. strains.

The MIC values of the selected commercial poultry products against the tested bacterial strains are summarized in Table 2, Figure 2 and Figure 3.

### 3.1. Gram-Negative Bacteria

#### 3.1.1. *Campylobacter* spp.

The MIC value of Herbal acid and Rigosol-N for *C. jejuni* S1 was 0.071% *v*/*v*. The MIC values for Eubisan 3000, Intesti-Flora, and Salgard^®^ liquid were 0.118% *v*/*v*, 0.284% *v*/*v*, and 0.568% *v*/*v*, respectively. Furthermore, the MIC values for Phyto CFC Liquide B, AEN 350 B Liquid, and ProPhorce^TM^ SA Exclusive against *C. jejuni* S1 were 0.142% *v*/*v*.

The MIC value for AEN 350 B Liquid, ProPhorce^TM^ SA Exclusive, Herbal acid, and Rigosol-N against *C. coli* S1 was 0.071% *v*/*v*. Moreover, the MIC value for Phyto CFC Liquide B, Intesti-Flora, and Eubisan 3000 was 0.142% *v*/*v*. The MIC value for Salgard^®^ liquid against *C. coli* S1 was 0.568% *v*/*v*.

The MIC value of AEN 350 B Liquid and Herbal acid was 0.071% *v*/*v*, whereas the MIC value of Phyto CFC Liquide B, ProPhorce^TM^ SA Exclusive, and Rigosol-N was 0.142% *v*/*v* for *C. jejuni* S2. The MIC values for Eubisan 3000, Intesti-Flora and Salgard^®^ liquid against *C. jejuni* S2 were 0.118% *v*/*v*, 0.284% *v*/*v*, and 0.568% *v*/*v*, respectively.

#### 3.1.2. *Escherichia coli*

The MIC value of ProPhorce^TM^ SA Exclusive, Herbal acid, and Rigosol-N against *E. coli* ATCC 25922 was 0.142% *v*/*v*. Furthermore, the MIC values for Eubisan 3000, Intesti-Flora, and Phyto CSC Liquide B were 0.379% *v*/*v*, 0.568% *v*/*v* and 1.894% *v*/*v*, respectively. The MIC value for AEN 350 B Liquid and Salgard^®^ liquid against *E. coli* ATCC 25922 was 1.136% *v*/*v*.

The MIC values for ProPhorce^TM^ SA Exclusive, Phyto CSC Liquide B, Salgard^®^ liquid, and AEN 350 B Liquid against *E. coli* ATCC 11303 were 0.071% *v*/*v*, 0.379% *v*/*v*, 0.568% *v*/*v* and 1.136% *v*/*v*, respectively. Moreover, the MIC value of Herbal acid and Rigosol-N was 0.142% *v*/*v*, whereas that of Intesti-Flora and Eubisan 3000 was 0.284% *v*/*v* for *E. coli* ATCC 11303.

#### 3.1.3. *Salmonella* Typhimurium

The MIC value of ProPhorce^TM^ SA Exclusive, Herbal acid, and Rigosol-N for *S.* Typhimurium DT120 was 0.142% *v*/*v*. The MIC values for Eubisan 3000, Intesti-Flora, and Phyto CSC Liquide B were 0.284% *v*/*v*, 0.568% *v*/*v* and 1.894% *v*/*v*, respectively. The MIC value for Salgard^®^ liquid and AEN 350 B Liquid against *S.* Typhimurium DT120 was 1.136% *v*/*v*.

The MIC values for ProPhorce^TM^ SA Exclusive, Eubisan 3000, and Intesti-Flora against *S.* Typhimurium U292 were 0.095% *v*/*v*, 0.284% *v*/*v* and 0.568% *v*/*v*, respectively. The MIC value of Herbal acid and Rigosol-N against *S.* Typhimurium U292 was 0.142% *v*/*v*, while Phyto CSC Liquide B, Salgard^®^ liquid, and AEN 350 B Liquid had MIC values of 1.136% *v*/*v*.

### 3.2. Gram-Positive Bacteria

#### 3.2.1. *Staphylococcus aureus*

The MIC value of ProPhorce^TM^ SA Exclusive, and Rigosol-N for *S. aureus* DSM 102262 was 0.142% *v*/*v*. Moreover, the MIC values for Eubisan 3000 and Intesti-Flora against *S. aureus* DSM 102262 were 0.568% *v*/*v*. The MIC values for Herbal acid, Salgard^®^ liquid, AEN 350 B Liquid and Phyto CSC Liquide B were 0.284% *v*/*v*, 1.136% *v*/*v*, 2.273% *v*/*v* and 9.090% *v*/*v*, respectively.

The MIC value of ProPhorce^TM^ SA Exclusive, and Rigosol-N for *S. aureus* DSM 25629 was 0.142% *v*/*v*. Furthermore, the MIC values for Intesti-Flora, Herbal acid and Eubisan 3000 against *S. aureus* DSM 25629 were 0.284% *v*/*v*. The MIC values for Salgard^®^ liquid, AEN 350 B Liquid and Phyto CSC Liquide B were 1.136% *v*/*v*, 4.545% *v*/*v* and 9.090% *v*/*v*, respectively.

Herbal acid and Rigosol-N had a MIC value of 0.142% *v*/*v* for *S. aureus* S1. Moreover, the MIC values for Intesti-Flora, ProPhorce^TM^ SA Exclusive, Eubisan 3000, and Phyto CSC Liquide B against *S. aureus* S1 were 0.237% *v*/*v*, 0.284% *v*/*v*, 0.758% *v*/*v* and 3.030% *v*/*v*. The MIC value for Salgard^®^ liquid and AEN 350 B Liquid was 1.894% *v*/*v*.

#### 3.2.2. *Listeria* spp.

The MIC value of ProPhorce^TM^ SA Exclusive, Herbal acid, and Rigosol-N for *L. monocytogenes* Scott A was 0.142% *v*/*v*. The MIC values for Salgard^®^ liquid and Intesti-Flora were 0.568% *v*/*v*. The MIC values for Eubisan 3000, AEN 350 B Liquid, and Phyto CSC Liquide B against *L. monocytogenes* Scott A were 0.379% *v*/*v*, 1.894% *v*/*v* and 2.273% *v*/*v*, respectively.

The MIC values for ProPhorce^TM^ SA Exclusive, Herbal acid, Rigosol-N, and Phyto CSC Liquide B against *L. innocua* ATCC 33090 were 0.142% *v*/*v*, 0.284% *v*/*v*, 0.379% *v*/*v* and 3.030% *v*/*v*, respectively. Moreover, the MIC values for AEN 350 B Liquid, Salgard^®^ liquid, Intesti-Flora and Eubisan 3000 against *L. innocua* ATCC 33090 were 1.136% *v*/*v*.

The MIC values for ProPhorce^TM^ SA Exclusive, Rigosol-N, Herbal acid, Eubisan 3000, AEN 350 B Liquid and Salgard^®^ liquid against *L. monocytogenes* S1 were 0.095% *v*/*v*, 0.118% *v*/*v*, 0.142% *v*/*v*, 0.189% *v*/*v*, 0.284% *v*/*v* and 1.136% *v*/*v*, respectively. Furthermore, the MIC value for Phyto CSC Liquide B and Intesti-Flora against *L. monocytogenes* S1 was 0.568% *v*/*v*.

Phyto CFC Liquid B altered the pH of the culture medium from 7.25 to 7.47 units during higher tested concentrations but did not change the pH in its lowest concentrations. The pH of the culture medium for the MICs at which Phyto CFC Liquid B was effective against the tested bacterial strains ranged from 7.25 to 7.47 units. AEN 350 B Liquid changed the pH of the growth medium from 7.10 to 7.47 units at the concentrations studied. The pH of the culture medium for the MIC values at which AEN 350 B Liquid was effective against the investigated bacterial strains ranged between 7.19 and 7.47 units. Furthermore, Salgard^®^ liquid decreased the pH of the culture medium at the tested concentrations from 4.58 to 7.43. The pH of the growth medium at which the specific product was able to inhibit bacterial growth for all the tested strains was between 5.02 and 5.30. Intesti-Flora altered the pH of the culture medium from 2.92 to 7.41 units during the tested concentrations. The pH of the culture medium for the MICs at which Intesti-Flora was effective against the tested bacterial strains ranged from 4.43 to 6.79 units. Moreover, ProPhorce^TM^ SA Exclusive changed the pH of the growth medium from 3.12 to 7.40 units at the concentrations studied. The pH of the culture medium for the MIC values at which ProPhorce^TM^ SA Exclusive was effective against the investigated bacterial strains ranged between 3.97 and 4.35 units. Herbal acid decreased the pH of the culture medium at the tested concentrations from 2.07 to 7.39. The pH of the growth medium at which the specific product was able to inhibit bacterial growth for all the tested strains was between 4.27 and 5.93 units. Rigosol-N changed the pH of the growth medium from 2.55 to 7.39 units at the concentrations studied. The pH of the culture medium for the MIC values at which Rigosol-N was effective against the investigated bacterial strains ranged between 4.91 and 5.73 units. Eubisan 3000 decreased the pH of the culture medium at the tested concentrations from 3.33 to 7.42 units. The pH of the culture medium for the MIC values at which Eubisan 3000 was effective against the investigated bacterial strains ranged between 4.77 and 6.95 units. Finally, the negative control wells had the same pH at all tested concentrations. The pH values of the dilutions of the commercial products in the appropriate medium are presented in Table 3.

## 4. Discussion

Antimicrobial resistance has reduced the effectiveness of various antibiotic classes and led the poultry sector to search for alternative compounds to control major poultry pathogens. The widespread adoption of natural alternatives such as phytogenic additives and acid-based eubiotics is advised because they are safe for the ecosystem, promote antibacterial activity, reduce the prevalence of antibiotic resistance aid in the production of organic chicken meat and are safe for human consumption [40]. Additionally, in the last decades, numerous in vitro studies demonstrated the efficacy of these compounds against poultry pathogens. However, the majority of these studies have focused on investigating the antimicrobial activity of individual essential oils or organic acids, with only a few examining the antibacterial activity of commercial products incorporating blends of the aforementioned categories [41,42,43].

Poultry serves as the primary reservoir for thermotolerant *Campylobacter* spp., with poultry meat being the primary vehicle for transmitting these microorganisms to humans [44]. The European Food Safety Authority (EFSA) has suggested that incorporating feed and/or water additives containing organic acids and/or essential oils during poultry production could reduce the prevalence of *Campylobacter*-positive flocks, thereby reducing the risk of human infection [45]. However, there is limited data available regarding the anti-*Campylobacter* activity of commercially available products. In the present study, all the products exhibited anti-*Campylobacter* spp. activity in concentrations ranging from 0.071% to 0.568% *v*/*v*. Particularly, Herbal acid, a product with large concentrations of lactic and phosphoric acids, also containing thyme and oregano essential oils, demonstrated efficacy by inhibiting the growth of the tested *Campylobacter* strains at a rather low concentration (0.071% *v*/*v*). This high efficacy of Herbal acid may be attributed to the synergic anti-*Campylobacter* activity of oregano oil and lactic acid, as previously reported by Navarro et al., 2015 [46]. Furthermore, the main active compounds in oregano and thyme oils, carvacrol and thymol, respectively, are known for their anti-biofilm properties and their ability to inhibit bacterial motility, as well as to alter bacterial cell membrane permeability, ultimately leading to cell death [32,47,48,49,50].

In contrast, Salgard^®^ liquid, a product rich in ammonium formate, required the highest concentration (0.568% *v*/*v*) among all tested products to inhibit the growth of *Campylobacter* spp. This finding is consistent with an in vitro antimicrobial efficacy investigation of sodium formate against *C. jejuni*, which revealed low antimicrobial activity [31]. *Campylobacter* species grow at an optimal pH range of 6.5–7.5, with survival even at pH levels close to 5.5 [51]. This could explain why Salgard^®^ liquid required such a high concentration to inhibit the growth of the tested strains, as in this concentration, the medium was under a pH of 5.3, which could be challenging for the growth of the microorganism, according to previous studies [31,51]. However, it is worth noting that despite its in vitro performance, Salgard^®^ liquid demonstrated efficacy in vivo when applied continuously in the drinking water of experimentally challenged by *C. jejuni* broiler chicks by reducing *C. jejuni* counts in the ceca of birds by 0.7log_10_ CFU/g [52].

Organic acid salts, such as those found in Salgard^®^ liquid, offer several advantages over free organic acids. They are less expensive, easier to handle in diets, less corrosive for the equipment, odorless to birds, and more water-soluble [53]. Additionally, salts can effectively bypass the stomach and undergo free acid forms in the lower parts of the gastrointestinal tract (GIT), whereas free organic acids are often metabolized and absorbed in the upper GIT of birds [54]. However, a major disadvantage of salts is that when the H+ ion is replaced by other cations, such as NH+, they do not lower the environmental pH to the same extent as free organic acids [52].

*Escherichia coli* is an omnipresent intestinal bacteria in humans and animals, which commonly develops antimicrobial resistance (AMR), further increasing its pathogenicity and highlighting the importance of exploring alternative antimicrobial agents. In the present study, all the tested commercial products recorded MIC values ranging from 0.071% to 1.894% *v*/*v* against the two strains of *E. coli* used (ATCC 25922, ATCC 11303). Particularly, Rigosol-N, a product based on oregano oil and lactic and acetic acids, exhibited antimicrobial activity against both tested strains of *E. coli* in the concentration of 0.142% *v*/*v*. Carvacrol, the primary active component of oregano oil, has been demonstrated to be an efficient agent against the growth of *E. coli* [55]. The antibacterial action of carvacrol against *E. coli* has been attributed to the downregulation of virulence genes, such as *stx* genes encoding Shiga toxin production [56]. Furthermore, the hydrophobic nature of carvacrol and other essential oils’ components allows them to interfere with bacterial lipid membrane synthesis, distributing enzymes and proteins and increasing membrane permeability, ultimately leading to bacterial cell death [57]. Moreover, acetic acid, in addition to lactic acid (both concentrated in Rigosol-N), is widely used in the food sector to reduce or control pathogenic bacteria, including *E. coli* [58,59]. It has been demonstrated that both lactic and acetic acids induce alterations in the metabolic processes of the bacteria [60]. A study found that a blend of lactic and acetic acid had a strong inhibitory effect on the biological processes within the cytoplasm of *E. coli* O157:H7 [61]. Additionally, a study reported that the combination of acetic acid and oregano oil inhibited the growth of *E. coli* O157:H7 on fresh lettuce [62].

Intesti-Flora exhibited higher MIC values (ranging from 0.284% to 0.568% *v*/*v*) for the tested strains of *E. coli* compared to other commercial products. Intesti-Flora is composed of both short-chain fatty acids (SCFAs), such as propionic and lactic acid, as well as medium-chain fatty acids (MCFAs), like sorbic acid. Additionally, it contains copper chelates and oligofructose syrup ingredients aimed at enhancing its effectiveness [33]. Propionic acid is known for its potent inhibitory effects against bacteria, yeasts, and molds [28]. The proliferation of the most pH-sensitive bacteria, such as *E. coli*, *Salmonella* spp., and *C. perfringens*, is minimized below a pH value of 5.0, whereas acid-tolerant bacteria survive [53]. To that end, a correlation between the antimicrobial activity of the specific product and the pH of the culture medium was noted. Specifically, the pH of the culture medium ranged between 5.0 and 5.5 units in the MICs, which is the product exhibited against *E. coli*. As a result, the use of acid-based eubiotics to lower pH, as attributed to the undissociated forms of the acids, may exhibit a direct inhibitory effect on bacteria. Acid-based eubiotics can easily cross the bacterial cell membrane and enter the cytoplasm, lowering intracellular pH, disrupting the cell membrane, and inhibiting enzymatic processes and nutrient transport systems [53].

Foodborne zoonotic *Salmonella* infections remain a major public health concern worldwide. Therefore, global efforts are required to decrease *Salmonella* surveillance, targeting poultry, which consists of a major natural reservoir [63]. Blends of essential oils and organic acids have gained interest in the last decades due to their synergic effect on growth performance, intestinal health, and antimicrobial activity [29]. Eubisan 3000, a blend of oregano, cinnamon, aniseed oil and citric acid, exhibited notable antibacterial efficacy against both strains of *S*. Typhimurium, with MIC values of 0.284% *v*/*v*. Previous studies have reported the in vitro antibacterial activity of compounds contained in Eubisan 3000 against several pathogenetic bacteria [64,65,66]. Two in vivo experiments utilizing combinations of organic acids and EOs in the nutrition of *S.* Enteritis-infected broiler chicks reduced *Salmonella* load in the ceca [67,68]. The primary mechanism underlying this synergism appears to be that hydrophobic molecules, such as EOs, increase the permeability of the bacterial cell membrane, allowing organic acids to permeate into the cells and alter the regulated efflux/influx of the bacteria cell wall. On the other hand, organic acids lower the intracellular bacterial pH, increasing the hydrophobicity of Eos, allowing them to penetrate the cell wall more easily and dissolve in the lipids, causing alterations in the bacterial membrane rigidity and fluidity [69].

*Staphylococcus aureus* is a widespread commensal and opportunistic pathogen found in humans and animals. In our study, all tested commercial products demonstrated inhibitory activity against the tested *S. aureus* strains, recording MICs varying from 0.142% to 9.090% *v*/*v*. Notably, Phyto CSC Liquide B, a highly concentrated blend of *trans*-cinnamaldehyde (the primary compound of cinnamon oil) and thymol (the primary compound of thyme oil), exhibited the highest MIC values against *S. aureus* strains in concentrations ranging from 3.030% to 9.090% *v*/*v*. This result could be ascribed to the biofilm formation of *S. aureus*, which makes the bacterium resistant to essential oils or to the low solubility and limited volatility of EOs [70].

*Listeria* spp., including pathogenic species like *L. monocytogenes*, pose a significant risk to human health due to their presence in raw and processed foods, as well as their ability to survive in various stress conditions (sanitization, pH, water activity, temperature), by forming biofilms on surfaces [71]. In this study, all the tested products demonstrated inhibitory efficacy against all the strains of *Listeria* spp. recording MICs ranging from 0.095% to 3.030% *v*/*v*. Particularly, ProPhorce^TM^ SA Exclusive, a blend of formic acid and sodium formate, demonstrated potent inhibitory activity against all strains of *Listeria* spp. at relatively low concentrations (0.095% to 0.142% *v*/*v*). The effectiveness of ProPhorce^TM^ SA Exclusive may be attributed to its ability to lower the pH of the broth medium in its specific concentrations, ranging from 4.0 to 4.3 units (Table 3), which is less than the growth limit of *Listeria* spp., which is 4.4 units [72]. Previous in vitro and in vivo studies have also reported the high antibacterial activity of ProPhorce^TM^ SA Exclusive against a variety of bacteria [31,52]. Formic acid and its salts, both concentrated in ProPhorce^TM^ SA Exclusive, are SCFAs. The capacity of SCFAs to lower pH has been reported to be determined by chemical parameters, such as the acid constant (pKa), dissociation constant (Ka) numbers, undissociated form concentration, and organic acid form concentration. Thus, the lower the pKa value, the more successful the acid is at lowering the pH of the medium. Most of the acids used as feed additives have pKa values ranging from 3.0 to 5.0 [53]. On the other hand, AEN 350 B Liquid, which is highly concentrated in *trans*-cinnamaldehyde, required relatively higher concentrations (0.284–1.894% *v*/*v*) to inhibit the growth of *Listeria* spp. strains. Previous studies have also reported the antimicrobial activity of *trans*-cinnamaldehyde against *Listeria* spp. [73,74]. However, research indicates *L. monocytogenes* can modulate its membrane fatty acid content to counteract the membrane fluidizing effect of *trans*-cinnamaldehyde [75].

Our investigation demonstrated that commercial essential oil-based phytogenic products, such as Phyto CSC Liquide B and AEN 350 B Liquid, exhibited higher MIC values against the gram-positive bacteria than gram-negative bacteria. This finding is aligned with previous in vitro studies that evaluated phytogenic natural compounds against bacteria [76,77,78]. The higher resistance of gram-positive bacteria to phytogenic products might be attributed to their thicker peptidoglycan coating cell wall, which restricts essential oils from entering the bacterial cell. Furthermore, in normal conditions, bacteria cells have a negative surface charge due to the presence of anionic groups (e.g., carboxyl and phosphate) in the membrane. A study demonstrated that gram-positive bacteria were more resistant to the reduction of the negative surface charge than gram-negative bacteria following exposure to EO compounds [77]. However, most research suggests that EOs and their components are more effective against gram-positive bacteria, which are mostly composed of a peptidoglycan layer. Gram-negative bacteria, on the other hand, have an outer membrane (OM) that contains hydrophilic lipopolysaccharides (LPS), preventing hydrophobic molecules such as EOs from penetrating the cell wall [69]. Finally, it must be highlighted that the comparison of the results of different studies is often difficult due to the use of different approaches, inoculum preparation techniques, inoculum size, growth medium, incubation conditions, and determination of endpoints.

The results of our study also showed a substantial strain-to-strain variability from some tested bacteria, such as *Listeria* spp., *S. aureus* and *E. coli*. In a previous investigation that included 62 clinical and food-associated *L. monocytogenes* isolates, several moderate differences in virulence and stress-associated genes were observed between the strains [79]. In addition, it has been suggested that the type of strain, the origin and the environmental conditions may impact the level of biofilm production by *L. monocytogenes* independently of the planktonic growth rate [80]. On the other hand, the genotypic and phenotypic characteristics of *S. aureus* strains may be responsible for the variability in biofilm formation [81]. Furthermore, a wide range of virulence pathways related to infectivity, toxin generation and antimicrobial resistance are associated with the strain variation of the bacteria [82].

## 5. Conclusions

The MIC values of eight commercial products commonly used in the poultry industry for water application against major zoonotic poultry pathogens, including *Campylobacter* spp., *Escherichia coli*, *Salmonella* Typhimurium, *Staphylococcus aureus*, and *Listeria* spp. were investigated. Our results indicate that all the tested products exhibited antimicrobial activity against the majority of the pathogens investigated. Notably, the products categorized as “Blends of essential oils and organic acids” (ProPhorce^TM^ SA Exclusive, Herbal acid, Rigosol-N and Eubisan 3000) demonstrated higher antimicrobial activity. Following closely were products classified as “Acid-based eubiotics” (Salgard^®^ liquid, Intesti-Flora), while products within the “essential oil-based phytogenics” category (Phyto CSC Liquide B, AEN 350 B Liquid) displayed the lowest antimicrobial capacity, against the tested pathogens.

Our in vitro findings shed light on the potential of natural antimicrobials as safe alternatives for preventing major zoonotic poultry pathogens and combating the emergence and spread of antimicrobial resistance. While our investigation provides promising insights, further research remains crucial to fully comprehend the mechanisms of action of these natural antimicrobials. Standardization of their effects on the health, welfare and performance of birds are essential avenues for future inquiry. Combinations of phytogenic additives and acid-based eubiotics emerge as particularly promising strategies, potentially surpassing the effectiveness of individual components akin to antibiotics. Leveraging optimal blends alongside impeccable management and husbandry practices will be paramount in reducing antibiotic usage in the poultry industry while maintaining or even enhancing production outcomes.

## Figures and Tables

**Table 1:** Information on the synthesis and the recommended dose of the selected commercial products.

Category	Tested Product	Active Ingredients	r. Dosage Range ^1^
*Essential oil*-based phytogenics	Phyto CSC Liquide B	*Trans*-cinnamaldehyde (43.93%), Thymol (29.83%), Carvacrol (10.56%)	0.030–0.060%
	AEN 350 B Liquid	*Trans*-cinnamaldehyde (87.12%), Eugenol (10.83%), (*E)*-caryophyllene (1.40%)	0.030–0.100%
Acid-Based Eubiotics	Salgard^®^ liquid	Ammonium formate (20%), Propionic acid (5.2%), Ammonium Propionate (1%) and Carrier	0.100–0.200%
	Intesti-Flora	Lactic acid, Propionic acid, Sorbic acid Copper-chelates of glycine, Oligofructose syrup	0.020–0.100%
Blends of essential oils and organic acids	ProPhorce^TM^ SA Exclusive	Formic acid (50–60%), Sodium formate (20–30%), L-(+)-lactic acid (5–10%), Cinnamaldehyde (1–5%)	0.080–0.100%
	Herbal acid	Lactic acid 30%, Phosphoric acid 20%, Formic acid 15%, Acetic acid 5%, Citric acid 1%, Malic acid 1%, Xtract anabasis (Thyme oil, Oregano oil) 0.5%	0.050–0.600%
	Rigosol-N	Lactic acid 60–80%, Acetic acid 20–10%, Propionic acid 5–10%, Benzoic acid 1–2%, Oregano oil 5%	0.040–0.060%
	Eubisan 3000	Oregano oil (80,000 mg), Cinnamon oil (3000 mg), Aniseed oil (3000 mg), Citric acid	0.020–0.025%

^1^: Recommended dosage range by the manufacturer (when animals are present).

**Table 2:** The minimum inhibitory concentrations (*v*/*v* *) of the tested commercial products on the selected bacterial strains (x ± SD).

	Phytogenics		Acid-Based Eubiotics		Blends of Essential Oils and Organic Acids			
Tested Strains	Phyto CSC Liquide B	AEN 350 B Liquid	Salgard^®^ Liquid	Intesti-Flora	ProPhorce^TM^ SA Exclusive	Herbal Acid	Rigosol-N	Eubisan 3000
	Gram-negative bacteria							
*C. jejuni* S1	0.142 ± 0.000	0.142 ± 0.000	0.568 ± 0.000	0.284 ± 0.000	0.142 ± 0.000	0.071 ± 0.000	0.071 ± 0.000	0.118 ± 0.041
*C. coli* S1	0.142 ± 0.000	0.071 ± 0.000	0.568 ± 0.000	0.142 ± 0.000	0.071 ± 0.000	0.071 ± 0.000	0.071 ± 0.000	0.142 ± 0.000
*C. jejuni* S2	0.142 ± 0.000	0.071 ± 0.000	0.568 ± 0.000	0.284 ± 0.000	0.142 ± 0.000	0.071 ± 0.000	0.142 ± 0.000	0.118 ± 0.041
*E. coli* ATCC 25922	1.894 ± 0.657	1.136 ± 0.000	1.136 ± 0.000	0.568 ± 0.000	0.142 ± 0.000	0.142 ± 0.000	0.142 ± 0.000	0.379 ± 0.164
*E. coli* ATCC 11303	0.379 ± 0.164	1.136 ± 0.000	0.568 ± 0.000	0.284 ± 0.000	0.071 ± 0.000	0.142 ± 0.000	0.142 ± 0.000	0.284 ± 0.000
*S.* Typhimurium DT120	1.894 ± 0.657	1.136 ± 0.000	1.136 ± 0.000	0.568 ± 0.000	0.142 ± 0.000	0.142 ± 0.000	0.142 ± 0.000	0.284 ± 0.000
*S.* Typhimurium U292	1.136 ± 0.000	1.136 ± 0.000	1.136 ± 0.000	0.568 ± 0.000	0.095 ± 0.041	0.142 ± 0.000	0.142 ± 0.000	0.284 ± 0.000
	Gram-positive bacteria							
*S. aureus* DSM 102262	9.090 ± 0.000	2.273 ± 0.000	1.136 ± 0.000	0.568 ± 0.000	0.142 ± 0.000	0.284 ± 0.000	0.142 ± 0.000	0.568 ± 0.000
*S. aureus* DSM 25629	9.090 ± 0.000	4.545 ± 0.000	1.136 ± 0.000	0.284 ± 0.000	0.142 ± 0.000	0.284 ± 0.000	0.142 ± 0.000	0.284 ± 0.000
*S. aureus* S1	3.030 ± 1.312	1.894 ± 0.657	1.894 ± 0.657	0.237 ± 0.082	0.284 ± 0.000	0.142 ± 0.000	0.142 ± 0.000	0.758 ± 0.328
*L. monocytogenes* Scott A	2.273 ± 0.000	1.894 ± 0.657	0.568 ± 0.000	0.568 ± 0.000	0.142 ± 0.000	0.142 ± 0.000	0.142 ± 0.000	0.379 ± 0.164
*L. innocua* ATCC 33090	3.030 ± 1.312	1.136 ± 0.000	1.136 ± 0.000	1.136 ± 0.000	0.142 ± 0.000	0.284 ± 0.000	0.379 ± 0.164	1.136 ± 0.000
*L. monocytogenes* S1	0.568 ± 0.000	0.284 ± 0.000	1.136 ± 0.000	0.568 ± 0.000	0.095 ± 0.041	0.142 ± 0.000	0.118 ± 0.411	0.189 ± 0.082

* MIC values are presented as %*v*/*v* concentration of the product in the Mueller-Hinton broth.

**Table 3:** pH values of culture medium used for the determination of the MICs according to the concentration of the commercial products examined.

Two-Fold Serial Dilutions	1	1/2	1/4	1/8	1/16	1/32	1/64	1/128	1/256	1/512	1/1024	1/2048
Phyto CSC Liquide B	7.25	7.30	7.35	7.39	7.45	7.46	7.47	7.47	7.47	7.47	7.48	7.48
AEN 350 B Liquid	7.10	7.19	7.28	7.37	7.42	7.46	7.47	7.47	7.47	7.47	7.47	7.47
Salgard^®^ liquid	4.58	4.63	4.74	5.02	5.30	6.20	7.00	7.21	7.31	7.37	7.41	7.43
Intesti-Flora	2.92	3.27	3.82	4.43	5.01	5.52	6.79	7.14	7.28	7.36	7.40	7.41
ProPhorce^TM^ SA Exclusive	3.12	3.23	3.39	3.58	3.81	3.97	4.00	4.35	5.10	5.90	6.60	7.40
Herbal acid	2.07	2.41	2.83	3.28	3.75	4.27	4.91	5.93	6.76	7.12	7.29	7.39
Rigosol-N	2.55	2.83	3.13	3.49	3.87	4.34	4.91	5.73	6.69	7.14	7.31	7.39
Eubisan 3000	3.33	3.78	4.27	4.77	5.39	6.35	6.95	7.14	7.31	7.37	7.42	7.42
Negative control ^1^	7.48	7.48	7.48	7.48	7.48	7.48	7.48	7.48	7.48	7.48	7.48	7.48

Grey coloring cells represent the two-fold serial dilutions at which each product inhibited the growth of the tested bacterial strains. ^1^: MH broth only.

## Data Availability

None of the data presented were deposited in an official repository.
